# Comprehensive Survey of Machine Learning Systems for COVID-19 Detection

**DOI:** 10.3390/jimaging8100267

**Published:** 2022-09-30

**Authors:** Bayan Alsaaidah, Moh’d Rasoul Al-Hadidi, Heba Al-Nsour, Raja Masadeh, Nael AlZubi

**Affiliations:** 1 Department of Computer Science, Prince Abdullah bin Ghazi Faculty of Information Technology and Communications, Al-Balqa Applied University, Salt 19117, Jordan; 2 Department of Electrical Engineering, Electrical Power Engineering and Computer Engineering, Faculty of Engineering, Al-Balqa Applied University, Salt 19117, Jordan; 3 Computer Science Department, The World Islamic Sciences and Education University, Amman 11947, Jordan

**Keywords:** augmentation, COVID-19, CT images, deep learning, diagnosis, machine learning, pneumonia

## Abstract

The last two years are considered the most crucial and critical period of the COVID-19 pandemic affecting most life aspects worldwide. This virus spreads quickly within a short period, increasing the fatality rate associated with the virus. From a clinical perspective, several diagnosis methods are carried out for early detection to avoid virus propagation. However, the capabilities of these methods are limited and have various associated challenges. Consequently, many studies have been performed for COVID-19 automated detection without involving manual intervention and allowing an accurate and fast decision. As is the case with other diseases and medical issues, Artificial Intelligence (AI) provides the medical community with potential technical solutions that help doctors and radiologists diagnose based on chest images. In this paper, a comprehensive review of the mentioned AI-based detection solution proposals is conducted. More than 200 papers are reviewed and analyzed, and 145 articles have been extensively examined to specify the proposed AI mechanisms with chest medical images. A comprehensive examination of the associated advantages and shortcomings is illustrated and summarized. Several findings are concluded as a result of a deep analysis of all the previous works using machine learning for COVID-19 detection, segmentation, and classification.

## 1. Introduction

COVID-19 is a new disease that surfaced two years ago. It is caused by Severe Acute Respiratory Syndrome Coronavirus 2 (SARS-CoV-2) [1]. The COVID-19 pandemic has affected health resources and most people’s lives—socially, educationally, and economically [2,3,4]. With the vast and growing number of infected people due to the fast spread of this virus, all countries have applied multiple measures to control the disease propagation. Most airports were closed, and governments assessed country-wide lockdowns; some cities were entirely quarantined as infested areas.

In addition to all the above complications, the COVID-19 diagnosis has many limitations and challenges of its own. For example, using PCR test kits incurs a considerable likelihood of false negatives, which is considered a primary drawback in diagnosis correctness. This leads doctors to use chest images (X-ray or CT scan) for a more accurate diagnosis that can better help doctors in their decisions. However, chest imaging availability is limited primarily due to the scarce availability of imaging equipment.

Producing a drug for COVID-19 takes months or even years due to the clinical trials that need to be carried out on humans and require approval for ethical and other reasons. Additionally, the various genetic mutations the virus shows cause further delay in producing a cure for this virus. From a technical perspective, many researchers have proposed and developed AI solutions for COVID-19 diagnosis, which helps the medical staff in their work, especially with the shortage of imaging equipment coupled with the large number of images needed for a more confident diagnosis by expert radiologists.

Machine Learning (ML) and deep learning are efficient tools that present solutions in medical image recognition and disease prediction [5,6,7,8]. Machine learning “is an interdisciplinary area, involving probability theory, statistics, approximation theory, convex analysis, algorithm complexity theory, and other disciplines” [9]. Machine learning algorithms are mathematical models used for prediction based on training these models using specific information.

Arthur Samuel proposed the “machine learning” concept for the first time in 1959 [10]. Since then, many machine learning algorithms have been developed based on a variety of mathematical concepts and are widely applied in daily life, such as biometric recognition [11,12], medical image recognition [13,14], speech and handwriting recognition [14,15], computer vision [16,17], aquaculture experiments [18,19,20,21], and robots [22,23].

Deep Learning or hierarchical learning concepts are a part of machine learning approaches based on data representation [24]. The deep learning methods rely on algorithms typically based on multi-layered neural networks corresponding to the abstraction levels [25]. The first model-based learning approach was introduced in 1965 by Oleksiy Ivakhnenko [26], who is named the “Father of Deep Learning” [27]. He presented a multilayer approach in which the input data properties are automatically filtered. The DL term was introduced in 1986 by Rina Dechter [28].

In DL, the most commonly used algorithm in image analysis is the Convolutional Neural Network (CNN) which is a multi-layer of perceptrons with minor pre-processing requirements [28,29]. The main difference between DL and traditional machine learning methods can be summarized by the feature extraction process [30]. The DL network structure is designed to extract the essential information using filters, as opposed to the traditional approach involving the feature engineering process, which is considered an expensive and challenging process [31,32,33].

One of the most significant challenges of the COVID-19 pandemic has been the highly contagious nature of the virus and elevated fatality rate. This increases the pressure on the health care system due to the massive number of cases that need a fast diagnosis. Combining the benefits of AI with medical knowledge can help the health system by providing automated solutions for COVID-19 diagnosis that can deal with many cases in a shorter time. Another advantage of using AI in COVID-19 diagnosis is reducing the human intervention required and consequently increasing the social distancing measures necessary to limit the infection’s spread.

The main challenge that may face ML in COVID-19 prediction is the limited availability of training data sets, as such sets have started with a relatively small number of images, despite being open-source data sets [34,35]. However, increasing the number of public data images available to researchers is expected to boost the accuracy rate of the trained ML systems.

Most of the artificial intelligence systems for COVID-19 detection are based on X-ray or CT scan images. However, some studies have been proposed based on blood tests for COVID-19 screening. Several features have been used in this type of detection models, such as gender, age, platelets, basophils, and monocytes, using different AI algorithms such as ANNs, SVM, KNN, decision trees, and random forest [36,37,38,39]. In this paper, the review focused on using image processing and machine learning algorithms for COVID-19 detection for X-ray and CT scan images.

Deep learning techniques that were proposed for COVID-19 classification have been presented and discussed in several previous review papers [40,41,42]. A more extensive survey was presented by Alyasseri et al. [43], in which the most efficient and highest-performance ML and DL systems were illustrated and explained. This study demonstrated and highlighted the proposed systems in specific publishers such as Elsevier, IEEE, and Springer. The studies were summarized according to which is related to ML or DL or a hybrid of both approaches.

This paper presents a comprehensive study of artificial intelligence systems applied for COVID-19 diagnosis using X-ray and CT (Computed Tomography) images which have been proposed, developed, and published, during the pandemic. This study covered the most popular studies regarding classification, segmentation, and data augmentation. Selection studies started using several sources, such as Google Scholar, Research Gate, and IEEE websites. The main keywords that were used for the search include AI, ML, DL, COVID-19, COVID-20, and segmentation. After collecting more than 200 papers, the studies are filtered according to the main subjects in which this study is organized.

This review paper consists of the following sections: Section 2 presents the research strategy of this paper. Section 3 presents the material that has been used in the reviewed systems. Section 4 discusses the proposed data augmentation techniques that have been used in several studies. Section 4 demonstrates and highlights the segmentation techniques of the infected areas of the lung tissues. Finally, Section 5 presents and illustrates the proposed classification system for COVID-19 detection.

## 2. Searching Strategy

**Figure 1:**
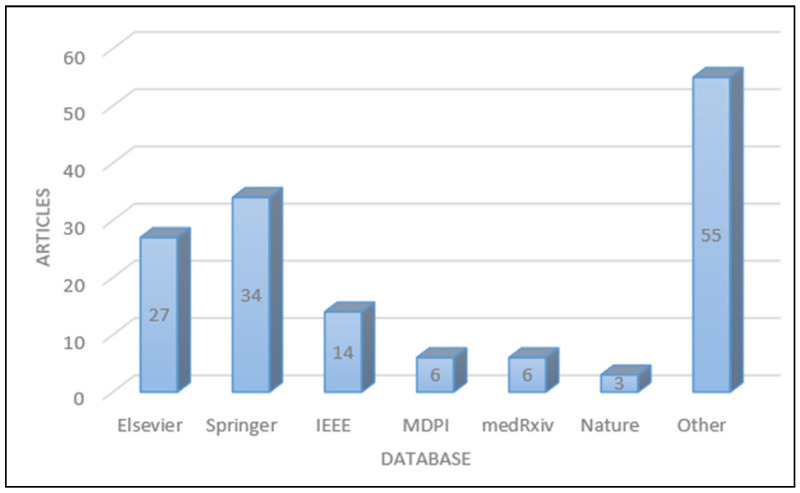
Number of articles per database.

The proposed survey started by searching process using the most used keyword such as: COVID-19, COVID-20, classification, machine learning, deep learning, artificial intelligence, segmentation, and data augmentation. Searching process has been carried out using several digital databases such as, Springer, Elsevier, MDPI, IEEE, medRxiv, nature, and others, as shown in Figure 1.

**Figure 2:**
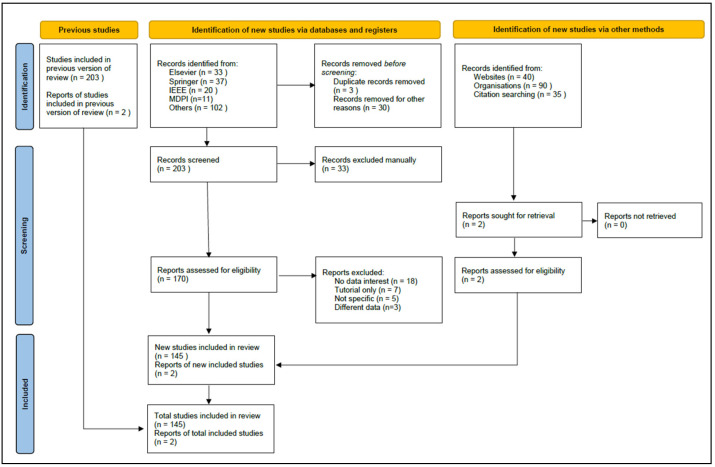
PRISMA flow chart.

Collecting articles was carried out in March 2022 which was the first step of the searching strategy, where 203 studies were retrieved using the mentioned keywords. The second step was screening the most relevant papers and excluding any duplicates or out of scope studies. There were 33 excluded articles, which present 16% of the total number of collected studies. According to the used keywords, several papers were filtered for eligibility in the third step of the proposed strategy. The papers should successfully combine COVID-19 or COVID-20 detection with machine learning or deep learning using chest images. Some articles presented a different type of database, use different techniques for diagnosis, or did not have the required information, such as the performance or the used dataset. Finally, 145 papers were extensively analyzed, illustrated, and summarized in this paper. The analysis covered the proposed systems including the performance and the limitations that can be improved by researchers. Figure 2 shows a summary of the selection strategy of the reviewed papers.

## 3. Materials

One leading limitation of the PCR test is the high rate of false-negative results, i.e., the diagnosis is negative, whereas the patient in reality is a positive carrier of the virus. Moreover, in many regions of the world, PCR test accessibility is restricted. Consequently, Computer Tomography (CT) and X-ray images can be used as the best alternative to identify this infection. CT or X-ray images are promptly accessible where there are no PCR test kits. Additionally, PCR kits are costly and take a lot of time to produce results, especially when the volume of tests is high. Furthermore, a professional clinician is needed to gather PCR tests, which may require additional training. Alternatively, it is somewhat simpler to work with CT and X-ray images.

Artificial Intelligence is a rising field that could fulfill a significant role in COVID-19 detection. For this purpose, any trained model needs enough data (mostly chest images) for virus recognition. With promising results, researchers have used machine learning to detect COVID-19 using medical images such as CT and X-ray images.

**Figure 3:**
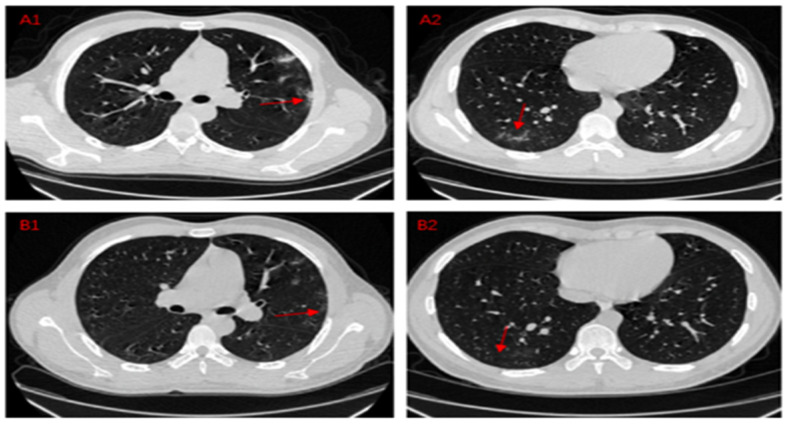
CT scan image of a COVID-19 pneumonia patient [36].

A CT image of the chest is taken using the computed tomography CT scan procedure. This procedure is known as computed axial tomography, a medical imaging technique that provides the clinician with detailed images of the body for diagnostic purposes [35]. Figure 3 shows a CT scan image of a COVID-19 pneumonia patient’s lung.

Despite its benefits, CT scans are generally considered expensive. Accordingly, clinicians use another type of chest image, namely X-ray images, instead of CT imaging. X-ray is a methodology that is traditionally used to produce chest images and is generally more available in most regions of the world, including developing countries. However, a CT scan generates images with further details, making COVID-19 diagnosis more effortless and efficient.

Several studies use CT scan images for COVID-19 detection. Yasar and Ceylan proposed a CNN model for COVID-19 detection using 386 CT scan images, and their study achieved 94.7% accuracy [44]. This is considered a low number of images, especially when using a deep learning algorithm. At the time of the study in 2020, the data set availability was limited.

In the following year (2021), the data sets had been increased because of the broader spread of the disease and the continuously growing number of patients. Since 2021, more studies have used CT scan images based on artificial intelligence algorithms for disease detection. For example, in [45,46,47], the researchers proposed artificial intelligence models for COVID-19 detection using CT scan images. The number of images in these studies was more significant than in the previous ones, and the system’s accuracy was 95.0, 93.44, 94.73, and 98.78%, respectively.

**Figure 4:**
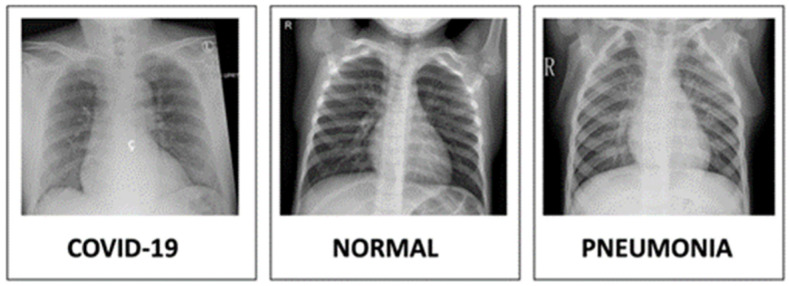
X-ray image examples for healthy and defected lungs [48].

Most studies have been conducted based on X-ray images. X-rays have much more limited frequencies than apparent light, which makes it conceivable to test structures significantly more modest than can be seen utilizing an ordinary magnifying instrument. This feature is utilized in X-ray microscopy to obtain high-resolution images and in crystallography to examine and find the location of atoms in precious stones [48]. Figure 4 shows an example of X-ray images.

X-ray images focus on bones and give more details about the surrounding tissues. Medical X-ray images are a critical product that uses radiation. In 1987, they represented 58% of human-made technologies in the United States. The more significant part came from traditional sources (82%); clinical X-rays represented just 10% of American radiation products [49].

By 2006, the operations in the United States were contributing significantly more ionizing radiation products than during the mid-1980s. In 2006, medical tools comprised almost 50% of the U.S.’s radiation products. This expansion reflects the advances and increased utilization of clinical imaging methodology, specifically processed tomography (CT) and atomic medicine [50].

Several organizations, such as Kaggle.com [51,52,53,54,55,56], github.com [57,58,59,60,61], and others, provide researchers with data sets of lung images like CT or X-ray images. According to different studies, some data sets are private and need access to be used. However, the public data sets have been rapidly and continuously growing.

Some studies used both CT and X-ray image types [62,63]. The first study used deep learning CNN for detection and classification and achieved the same accuracy for both CT and X-ray images. However, the second study proposed a different algorithm for the two types of images which gave different performances, where it was 93.44% for X-ray images and 87.98% for CT images.

## 4. Data Augmentation

Data augmentation is a great process that can give new, extra images that save the original information. However, it can likewise create noise that can affect the training process efficiency of the AI model. For example, data augmentation was necessary, especially at the beginning of the pandemic, as the number of images was negligible. At the same time, the artificial intelligence algorithms required a more extensive data set to be trained efficiently.

In the study [64], the authors used a data set with 585 X-ray images. This number is considered minor, and consequently, the researchers attempted to augment these images for a more efficient training experience. The images were produced randomly during the training process by flipping, translation, and rotation operations. Table 1 demonstrates the used augmentation algorithms.

**Figure 5:**
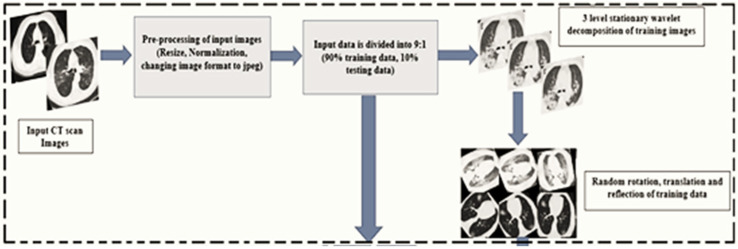
Data augmentation process as proposed in [65].

In another study [65], data augmentation was carried out by several transformation processes, as follows, and as shown in Figure 5:

- Use stationary wavelets to split the training images into three levels;
- Apply shear operation using values [−30, 30];
- Apply rotation transformation within [−90, 90];
- Translate the pixels within [−10, 10].

As indicated earlier, the data augmentation process is a useful tool, but it can produce noise that affects the training model’s efficiency. Nishio et al. combined three different methods for data augmentation to prevent the overfitting problem. They used the conventional method, mixup, and RICAP, with the following parameters [66]:

- ±15° rotation;
- ±15% *x*-axis shift;
- ±15% *y*-axis shift;
- horizontal flipping;
- 85–115% scaling and shearing;
- mixup = 0.1.

The results of this work showed better accuracy using a combination of the three algorithms than no-augmentation, or using any of the mentioned three methods separately [66]. Another study aimed to prevent the overfitting problem by monitoring the training loss. The data augmentation was done based on rotation and flipping [67].

**Figure 6:**
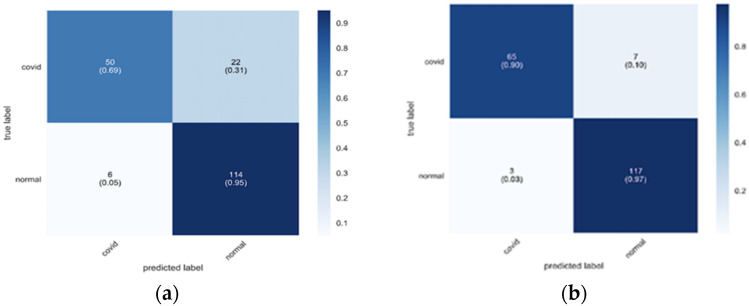
(**a**) With data augmentation; (**b**) without data augmentation [68].

The mentioned data augmentation techniques, such as flipping, rotating, and color changing, are fast, reliable, and easy to apply. However, such changes in the structure are limited in their benefits and do not produce completely new data. For example, in [68], the authors proposed a new technique for data augmentation using a Generative Adversarial Network (GAN). This model can generate artificial images with inconspicuous examples and without supervision [69]. The main idea of this network is to utilize two restricting networks and a generator that delivers a clear picture to deceive the other network that is prepared to most separate between the positive and false images in the discriminator [70]. Figure 6a,b shows the system performance with, and without, data augmentation [68].

## 5. Segmentation

In some studies, researchers have tried to segment the infected tissues of the lung to develop COVID-19 virus detection in CT scan images. The segmentation process can be considered a Region of Interest (ROI), which improves the system performance by specifying only the infected area and minimizing the wasted time reading all the image features.

Despite all the advantages of segmenting the infected lung tissues, there are several challenges facing this process, such as the high variation between infected cells, the irregularity of these cells, the low contrast of the tissues, and the differences in the illumination. Irrespective of the mentioned challenges, some studies proposed several algorithms for defected tissue segmentation. For example, in [71], the authors used Support Vector Machine (SVM) and active contour modeling to segment the defected tissues. The detection rate of three types of nodules (solid, non-solid, and cavitary) was 89%, whereas the false positive was 7.3%, and the proposed model correctly specified the location of the nodules.

In the study [72], three benchmarks were built for lung and infection segmentation processes using 70 COVID-19 cases, which contain momentum dynamic research field-stones, e.g., scarcely any shot learning, area speculation, and information move. This work was proposed using 40 pre-trained models. The results of this research were 67.3% for average Dice Similarity Coefficient (DSC) scores of infection segmentation, whereas the average Normalized Surface Dice (NSD) score was 70.0% for infection area segmentation.

**Figure 7:**
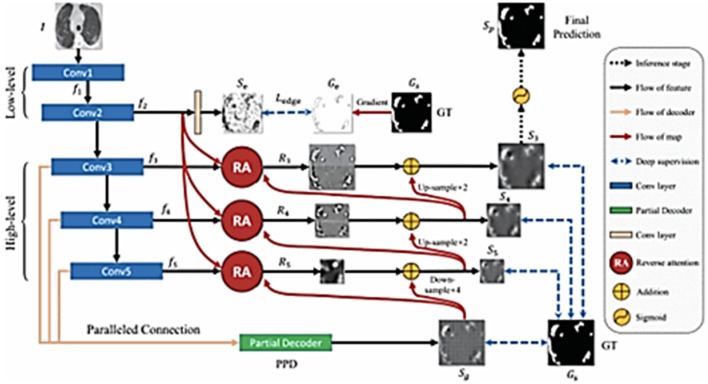
Infection Network (Inf-Net) for segmentation [73].

**Figure 8:**
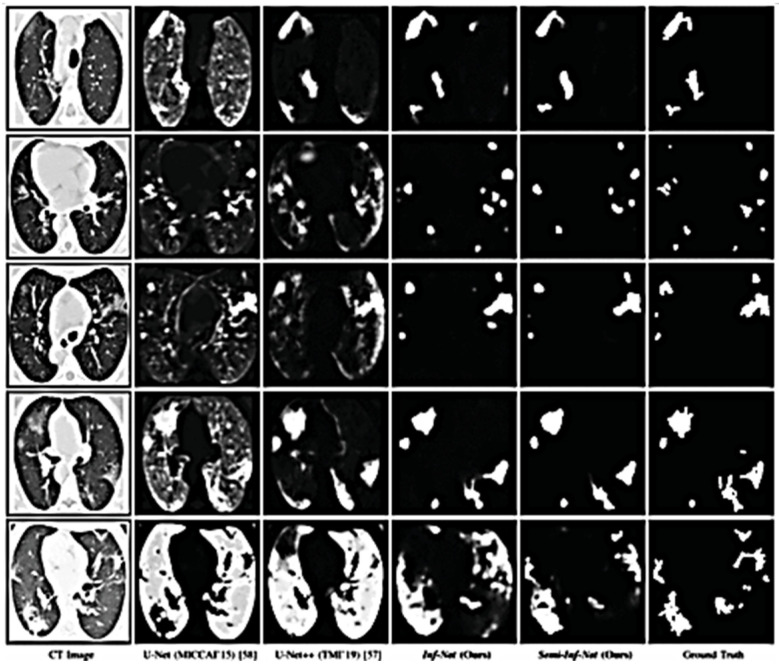
Inf-Net performance compared with others [73].

Another study to address the lung segmentation challenges was proposed by Fan et al. [73]. This study was conducted based on Infection Network (Inf-Net) for segmentation. This system started with extracting the low-level features. The performance of these features was increased by adding an edge attention model. The overall infection network is shown in Figure 7. The system performance was promising and considerably closer to the ground truth compared to other studies, as shown in Figure 8.

**Figure 9:**
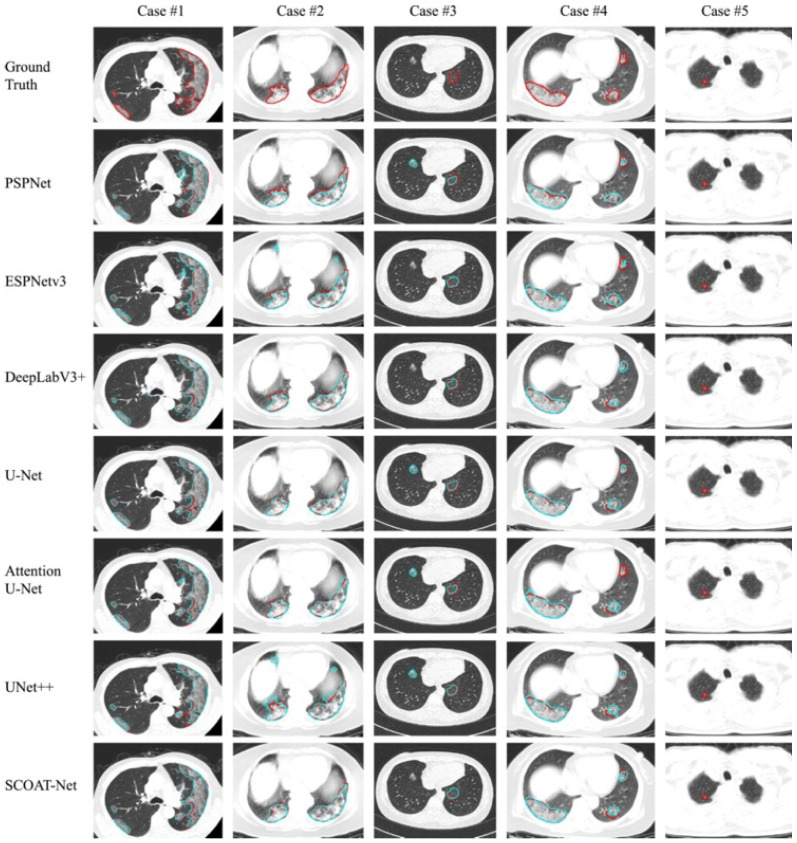
Segmentation performance of different models [74].

In another study, the authors used the UNet++ for lung segmentation by inheriting the basic structure of UNet++ and then composing it with SCOAT-Net based on the encoder and the decoder semantic level. This method uses a smaller number of parameters and reduces the calculation cost. Further, the performance was good for small-scale datasets. On the other hand, this method could not detect certain delicate opacity regions, as shown in case 5 in Figure 9 [74].

Based on the proposed literature, infected cell segmentation provides radiologists with more informative results related to the volume, shape, and percentage, of the infected area. Furthermore, this process generates the most important and impressive information for ML models, which has been validated in numerous studies [72,75,76,77].

**Figure 10:**
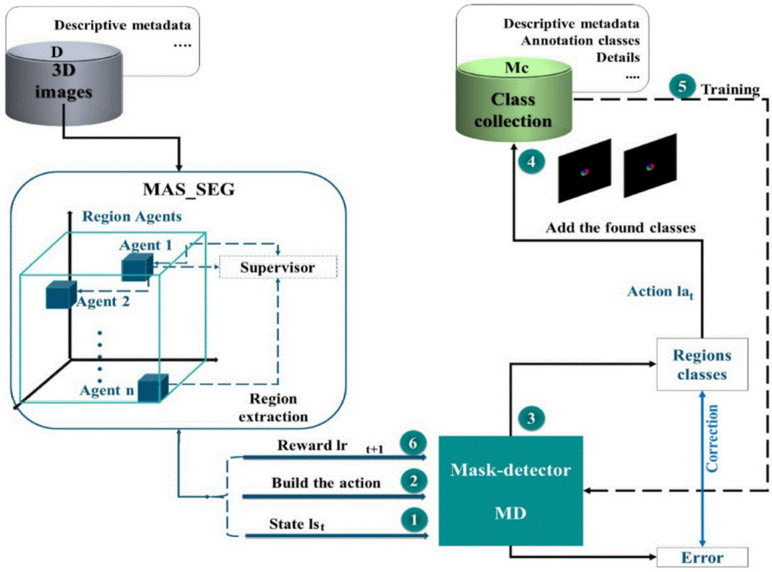
Architecture of 3D mask detection using MARL approach [78].

To minimize the time that has been consumed for manual masking of the infected tissues of the lung, an enhanced segmentation framework was introduced in [78]. A multi-agent Deep Reinforcement Learning (DRL) approach was proposed for lung infection segmentation, which is an improved version of Deep Q-network and based on CT images. The suggested mask detection was carried out like a tree for 3D images to cover all agents in terms getting a best segmentation and detection process, as shown in Figure 10. This study outcome was compared with the other existing segmentation systems and the ground truth; the precision of the proposed model was 97.12%, the sensitivity, specificity, precision, and the F1 score were 79.97, 99.48, 85.21, and 83.01%, respectively. This model used the multi-agent to get several masks for a 3D image to cover all areas and specially the unobservable regions of the lung.

## 6. Classification

Radiologists recently discovered that the DL approach, which was able to detect tuberculosis in chest X-rays, could also be practical for recognizing COVID-19-related lung abnormalities, and assisting physicians in choosing the treatment order for high-risk COVID-19 patients. Others have proven that medical imaging is an essential source of information for a quick diagnosis of COVID-19 and that the combination of AI and chest imaging can assist in explaining COVID-19 problems [76].

In terms of COVID-19 image analysis, a chest X-ray is an imaging tool used by hospitals to diagnose COVID-19 infection, and it was the first image-based strategy utilized in Spain. Suppose clinical suspicion of infection exists after inspection. In that case, a sample of nasopharyngeal exudate is taken to evaluate the reverse transcription-polymerase chain reaction (RT-PCR), followed by the acquisition of a chest X-ray film. Because the results of the PCR test can take several hours to obtain, the information revealed by the chest X-ray is critical for a quick clinical diagnosis. For example, if the patient’s clinical condition and chest X-ray are both normal, the patient is sent home while the etiological test results are awaited. The suspected patient will be admitted to hospital for close observation if the X-ray reveals abnormal results [77].

In general, the lack of, or presence of, abnormal signs on a chest X-ray is used to determine whether the patient should be sent home or kept in the hospital for additional observation. While radiography in medical examinations can be performed quickly and widely due to the prevalence of chest radiology imaging systems in healthcare systems, the radiologists’ ability to interpret radiography images is limited due to the human capacity to detect subtle visual features present in the images [79].

Many studies have been reported in this literature on new advances in DL models employing types of neural networks for separating COVID-19 from non-COVID-19 cases using neural networks, since AI can uncover patterns in chest X-rays that radiologists would generally miss.

COVID-19 and other kinds of pneumonia with different localization from chest X-rays are proposed to be detected quickly using a deep neural network architecture called CovXNet. Instead of utilizing classic convolution, efficient depth-wise convolution with varying dilation rates is used to analyze anomalies in 5856 X-rays images from various perspectives by integrating data from multiple receptive fields. For the initial training of the deep network, an enormous database containing X-rays from normal and other typical pneumonia patients is used to supplement the modest number of COVID-19 X-rays. Due to overlapping solid characteristics between COVID-19 and other pneumonia, a very excellent result may be achieved with a smaller database, including COVID-19 X-rays, by transferring the first trained convolutional layers with some extra fine-tuning layers [80].

Furthermore, it has been discovered that stacking algorithm improvements can be made by further improving predictions obtained from multiple CovXNet variants, which are principally optimized with varying input X-ray resolutions. In addition, a created class activation map allows for the discriminative localization of aberrant zones, which can aid in the diagnosis of clinical pneumonia symptoms on X-rays. More sample X-rays of COVID-19 patients for training in the transfer learning phase should increase the performance of these schemes even more.

Extensive simulation findings indicate that it could be an effective solution for the faster diagnosis of COVID-19 and other pneumonia patients. Furthermore, the proposed CovXNet is extremely scalable and has a sizeable receptive capacity, making it suitable for various computer vision applications. As a result, a gradient-based discriminative localization is implemented to identify the aberrant regions of X-ray pictures relating to distinct forms of pneumonia [78].

**Figure 11:**
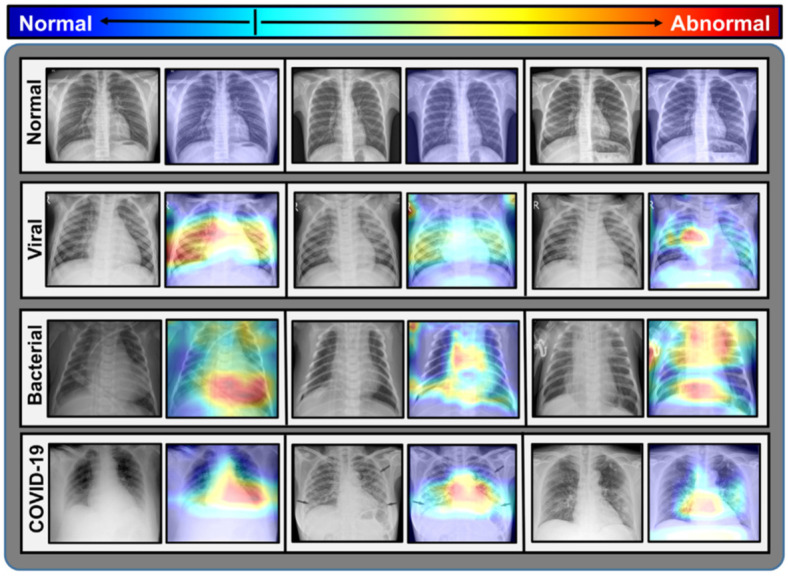
COVID-19 cases detection [78].

Extensive testing utilizing two distinct datasets reveals that COVID-19/Normal, 96.9% for COVID-19/Viral pneumonia, and 94.7% for COVID-19/Bacterial pneumonia. On the other hand, the accuracy was 90.2% for multiclass COVID-19/normal/Viral/Bacterial pneumonia, providing outstanding detection performance, shown in Figure 11. As a result, at the current state of the COVID-19 pandemic, the proposed methods can be an effective tool.

In another attempt, the authors present a model for automatically collecting the radiological abnormalities congruent with COVID-19 in chest CT data and creating a robust ML model [79]. These findings show that a typical machine learning approach can predict the presence of the virus in a radiological test. To improve the model, an AI system was utilized to extract COVID-19-related illnesses and feed them to the algorithm as supplementary data, with an accuracy of 89.15%. The best method proposed (SVM) is efficient, quality, and cost-effective. As a result, radiologists will use this approach as a decision support tool to detect suspected COVID-19 cases in real-world circumstances and attain roughly 90% accuracy with this method, improving the baseline findings by five points.

A multi-classification deep learning model for detecting COVID-19, pneumonia, and lung cancer, has been developed and tested from X-rays and CT scans of the chest. According to our knowledge, this model is accurate [80]. The first attempt is to classify the three chest disorders systematically. A single design is critical to accurately diagnose these conditions as soon as possible, as COVID-19 patients should receive the appropriate treatment and be isolated from halting the spread of the virus. There were four different architectures. VGG19+CNN, ResNet152V2, ResNet152V2+GRU, VGG19+CNN, ResNet152V2+GRU, VGG19+CNN, ResNet152V2+GRU, VGG19+CNNResNet152V2+Bi-GRU, and ResNet152V2+Bi-GRU.

Through extensive experiments and results performed on collected datasets from several sources that contained chest X-ray and CT images, the VGG19+CNN model outperformed the other three proposed models. The VGG19+CNN model achieved 98.05% accuracy, 98.05% recall, 98.43% precision, 99.5% specificity, 99.3% negative predictive value, 98.24% F1 score, 97.7% MCC, and 99.66% AUC, based on X-ray and CT images [80].

**Figure 12:**
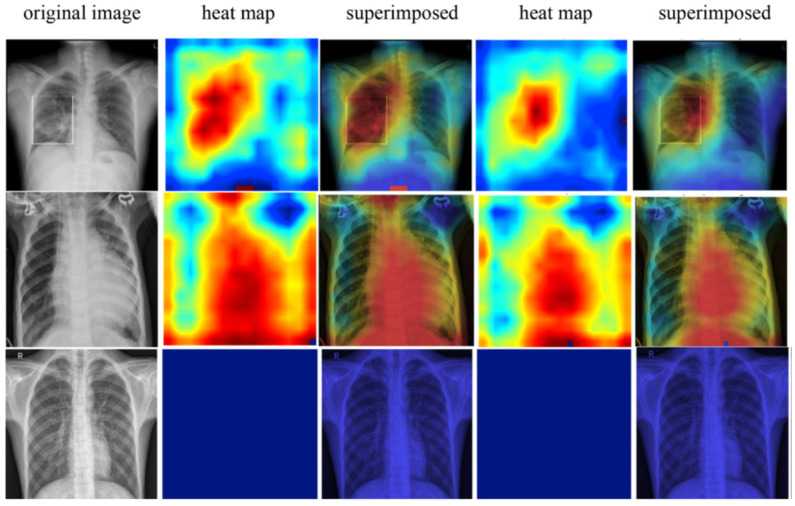
COVID-19 detection and diagnosis [81].

A Computer-Aided Design (CAD) technique for identifying COVID-19 patients from 2.300 CXR pictures is provided. A rich representation is built from an optimal set of GLCM-based texture features to precisely represent the segmented lung tissue ROIs of each CXR image [81]. The collected features are normalized for the final COVID-19 classification and fed into a discriminative LDCRF model. Using 5-folds cross-validation, the approach was thoroughly evaluated and validated on a large publicly available dataset of frontal CXR pictures, reaching an average accuracy of 95.88%, with precision, recall, and F1-score of 96.17%, 94.45%, and 95.79%, respectively. These findings show that the suggested CAD approach can assist radiologists and medical physicists in developing a reliable diagnosis model to differentiate COVID-19 from non-COVID-19, as shown in Figure 12.

There are three types of deep learning approach for classifying and segmenting X-ray pictures of COVID-19 virus-infected patients’ lungs. Two systems are presented for patient diagnosis: a deep neural network (DNN) method based on the fractal feature of input photos, and a CNN method based on CT scan images [82]. The given CNN architecture, with greater accuracy (93.2%) and sensitivity (96.1%), outperforms the DNN technique, which has an accuracy of 83.4% and a sensitivity of 86%. With impressive performance, the provided CNN architecture can be used to diagnose COVID-19 patients.

Reverse Transcription Polymerase Chain Reaction is currently the best method for diagnosing patients (RT-PCR). However, many people cannot use this method on patients; the procedure is costly and time-consuming. As a result, it is critical to propose an artificial intelligence strategy for better diagnosing COVID-19 patients. The resulting approach can be used in place of RT-PCR and demonstrates a reliable and successful approach to COVID-19 patient non-contact testing, which can aid in the early and cost-effective detection and screening of COVID-19 cases. To see if the model extracts enough biomarkers for COVID-19 positive cases, a group of medical specialists will have to work together [82].

The regions of focus for verified COVID-19 positive cases, bacterial pneumonia, and healthy cases, are shown in the CAM images of chest radiographs. Methods mentioned in this study could be used as an initial screening tool to assist healthcare providers in treating COVID-19 patients by better recognizing and promptly screening illness. In addition, it offers not only a low-cost but also high-quality service. In addition, medical practitioners can use an automatic noncontact testing approach to reduce the risk of contracting COVID-19. Initial tests revealed that the model produced satisfactory results and may be utilized to speed up COVID-19 identification. In two and three output class examples, the experimentation revealed an accuracy of 96% and 92.5% [82].

Based on chest X-ray pictures, the current study used three deep CNN techniques to detect COVID-19. Two transfer learning methodologies were evaluated: deep feature extraction and fine-tuning, as well as an end-to-end trained new CNN model. In addition, SVM classifiers were used to classify the in-depth features and several kernel functions. Analyzing eight well-known local descriptors yielded the following conclusions: Local descriptors outperformed deep learning algorithms [83]. Deep features and the SVM classifier exceeded the other approaches; deep feature extraction and local feature descriptor extraction take less time than fine-tuning and end-to-end training. In deep feature classification, the Cubic kernel function surpassed all other kernels.

The ResNet50 model outperformed the other pertained CNN models in most cases. Deep CNN models outperformed external networks in terms of end-to-end training. The accuracy score for the deep features retrieved from the ResNet50 model and SVM classifier using the Linear kernel function was 94.7%, the highest of all the findings. The fine-tuned ResNet50 model had a 92.6% success rate, whereas the constructed CNN model had a 91.6% success rate after end-to-end training. Various local texture descriptors and SVM classifications were also employed to compare performance with alternative deep approaches to identifying COVID-19 based on chest X-ray images. The findings indicated that the deep techniques tend to be efficient when compared to the local texture descriptors [83].

COVID-19 symptoms were diagnosed using Artificial Intelligence models based on human-generated respiratory sounds such as voice/speech, cough, and breath. The Convolutional Neural Network is a type of AI network used to manage various real-world challenges. The Deep Convolutional Neural Network (DCNN) model was used in this study to diagnose COVID-19 disease using human respiratory sounds from the COVID-19 sounds crowdsourced dataset [84].

Using multi-feature channels instead of standard techniques allowed the model to extract deep features of the acoustic respiratory sound signal with a 7% higher accuracy on the COVID-19 crowdsourced benchmark dataset. Using a DCNN classifier, the model classified sounds as asthma sounds, COVID-19 sounds, pertussis, bronchitis, and ordinary healthy sounds, with an accuracy of 95.45%. COVID-19 detection using respiratory sounds is the suggested approach to improve the efficiency of detecting COVID-19 positive cases.

Automatic detection of COVID-19 from chest X-ray pictures, EMCNet, can help affected patients. EMCNet extracts high-level characteristics from X-ray pictures using CNN. The ensemble model accurately identifies COVID-19 vs. normal instances. In comparison with a previous recent study, the dataset comprises a considerable number of COVID-19 photos with 98.91% accuracy, 100% precision, 97.82% recall, and 98.89% F1-score; a thorough trial reveals improved performance. With these findings, EMCNet can serve as a valuable resource for doctors and may be used as an alternative to manual radiological analysis for the automatic detection of COVID-19. EMCNet has certain limitations, for example, it can misclassify some COVID-19-positive situations as negative, but it can be used as a backup [84].

For the detection of new COVID-19 cases from X-ray pictures, a deep CNNLSTM network was introduced. For coronavirus detection, CNN is employed as a feature extractor and the LSTM network as a classifier. Combining retrieved characteristics with LSTM that distinguish COVID-19 cases from others improves the performance of the proposed system. The created system had a 99.4% accuracy, 99.9% AUC, 99.2% specificity, 99.3% sensitivity, and a 98.9% F1-score. On the same dataset, the proposed CNN-LSTM and competitive CNN architectures are used [85]. The results of the rigorous testing demonstrated that the proposed design outperforms a competing CNN network and that the suggested system could produce a tool for COVID-19 patients and caregivers during this global COVID-19 pandemic.

To lessen the effort of medical diagnosis, they employ X-ray pictures of the chest region collected from COVID-19 infected patients, as well as healthy people, to build a system using a deep transfer learning approach. Three CNN models, Inception- ResNetV2, InceptionV3, and ResNet50, are compared, with ResNet50 providing the best performance and accuracy of around 98% [86]. In the suggested framework, the class imbalance problem is solved utilizing a modified loss function and numerous layers of convolution and capsule. Following the adoption of this system, doctors and those in clinical practice would be able to make better decisions due to enhanced performance. In addition, the model’s area under the curve, specificity, and accuracy, can all be improved with pre-training. The number of images in the datasets can be increased to improve the quality.

Deep feature and SVM are used to adapt an approach for detecting coronavirus (COVID-19) using X-ray pictures by extracting the deep features of 13 pre-trained CNN models and feeding them to the SVM classifier one by one. Each classification model is run 20 times, and the average value is recorded to improve the robustness of the classification model [87]. The ResNet50 plus SVM classification model performs better than the other 12 classification models. The proposed classification model for COVID-19 detection has a 95.33% accuracy rate. The accuracy of 95.33% is based on the average of 20 independent executions, with a maximum accuracy value of 98.66%. A vast dataset might be used to expand this study. The method’s restriction is that it cannot be used if the patient is in pain.

Zhou et al. proposed a segmentation network based on the U-Net with an attention mechanism [88]. Because most contemporary segmentation networks are trained with dice loss, which equally penalizes false negative and false positive voxels, they contribute a high specificity but low sensitivity. They used the focused Tversky loss to train the model to improve the tiny ROI segmentation performance. Furthermore, they enhanced the baseline U-Net by adding the attention mechanism into each layer to capture rich contextual interactions and improve feature representations.

The outcomes of the experiment show that their proposed strategy is effective. However, the study is constrained by the tiny dataset. It believes that the proposed method could obtain more competitive outcomes with a more extensive training dataset. The experiment’s findings, evaluated on a short dataset with only 100 CT available slices, show that the proposed strategy works. On COVID-19 segments, it is possible to produce accurate and quick segmentation. The dice score, sensitivity, and specificity achieved are 69.1%, 81.1%, and 97.2% of the time, respectively [88].

Growing the number of research proposes employing deep learning to enable quick and dependable COVID-19 assessment using chest CT using a multi-center dataset. The study suggested the first systematic comparison of a wide range of deep learning approaches for CT segmentation. Seven in-house deep learning methods were compared to four public deep learning methods [89]. All procedures predicted overall lesion volume with an average volume deference that was lower than the human rater’s accuracy. In addition, they compare 12 deep learning methods using a multi-center dataset, including open-source and in-house built algorithms.

The results suggest that combining several approaches improves overall test set performance for lung segmentation, binary lesion segmentation, and multiclass lesion segmentation, with mean dice scores of 0.982, 0.724, and 0.469, respectively. The binary lesions were segmented with a mean absolute volume error of 91.3 mL. With a mean fundamental volume difference of 152 mL and mean dice scores of 0.369 for consolidation and 0.523 ground-glass opacity, identifying different lesion types was more difficult. All algorithms accomplish binary lesion segmentation with an average volume error lower than that of human raters’ visual assessment, implying that methods have matured to the point where they can be evaluated on a wide scale and used in clinical practice [89].

A deep learning approach for COVID-19 lung infection segmentation in chest CT scans was provided in this paper [90]. The FCN was designed utilizing a U-net architecture as the backbone, with proposed ResDense blocks at each level along the encoding and decoding routes. Because of the concatenation skip connection in each ResDense block, the feature maps of the infection zones and lung backdrop travel through the network with a minor change in their values, improving network learning and segmentation performance. Furthermore, the method includes an EED step that enhances the look of infection regions in CT slices by increasing contrast and homogeneity.

The qualitative and quantitative evaluation results demonstrate the system’s usefulness and ability to segment COVID-19 infection regions from CT images. This system is trained and verified using a variety of datasets from various sources, demonstrating its generalizability and potential as a tool for automatic COVID-19 infection segmentation and clinical practice. Researchers used different metrics for lung and infection regions segmentation and achieved dice overlapping scores of 0.961 and 0.780, respectively. Many 2D CT slices taken from various datasets from various sources are used to train and evaluate the proposed system, demonstrating its generality and efficiency [90].

COVID-19’s diagnostic performance was improved using pre-trained knowledge and study of the potential using a deep learning technique called returning transfer learning, to help clinicians diagnose COVID-19 [91]. COVID-19 was distinguished from viral and bacterial pneumonia by their approach. Their method can potentially increase the efficiency of diagnosis, isolation, and the treatment of COVID-19 patients, relieve radiologists’ workload, and bring the pandemic under control. Using ResNet in binary classification, the suggested system can accurately classify COVID-19 from healthy patients, COVID-19 from bacterial pneumonia, and COVID-19 from viral pneumonia. Using this model provided 97.20% accuracy on three classes and 80.95% on four classes in multi-class classification.

Automatic algorithms for classifying chest X-ray pictures using three separate categories of analysis: COVID-19, pneumonia, and healthy patients are presented [92]. Given the similarity in pathological impact on the lungs between COVID-19 and pneumonia, particularly during the early phases of both lung diseases, they conducted a comprehensive investigation of differences considering various pathological circumstances. They evaluated six representative state-of-the-art deep network architectures on three different public datasets to address these classification tasks: (I) the Radiological Society of North America (RSNA) Chest X-ray dataset; (II) the COVID-19 Image Data Collection; and (III) the SIRM dataset of the Italian Society of Medical Radiology.

Various typical tests were done to validate the designed methodologies for 6070 chest X-ray radiographs classification. In general, the outcomes of the experiments were favorable. The developed methods achieved accuracy values of 0.9706, assisting doctors in diagnosis and, as a result, allowing for early treatment of this crucial pandemic pathology [92].

A deep convolutional neural network is proposed to detect COVID-19 pneumonia patients using digital chest X-ray pictures, while maximizing detection accuracy (DCNN) [93]. The collection has 864 COVID-19, 1345 viral pneumonia, and 1341 normal chest X-ray images. In this study, a DCNN-based model called Inception V3, with transfer learning, was developed for detecting coronavirus pneumonia infected patients using chest X-ray radiographs, with a classification accuracy above 98% (training accuracy of 97% and validation accuracy of 93%). The findings reveal that transfer learning for COVID-19 detection is effective, has stable performance, and is simple to implement.

A Deep Convolutional Neural Network technique for identifying COVID-19 infection cases from patient chest X-ray pictures quickly and reliably is presented [94]. Chest X-ray pictures of more than 150 verified COVID-19 patients from the Kaggle data pool were used in the experiments to validate the performance of the proposed method. The results reveal that the suggested method correctly detects the results 93% of the time.

Based on the knowledge gained from CT scan images, a new COVID-19 diagnosis technique is developed as a binary classification challenge utilizing a sequential CNN. The findings indicate that the model is quite effective at doing its job, with a maximum accuracy of 92.48% [95]. Other linked parameters confirm the validity of the proposed method’s outcome and establish its superiority over earlier methods. The main goal of this research is to aid in the worldwide war against COVID-19, which medical professionals are waging with zeal, by providing them with a simple and effective method of diagnosing the condition. As academics, we hope that this approach will be applied and used globally and that we will be able to contribute to putting this pandemic behind us.

An integrated deep learning architecture for COVID-19 classification is presented using two widely used classification networks, ResNet and Xception, to run experiments to uncover obstacles and limitations [96]. The findings reveal that deep learning models can overestimate their performance due to experimental design flaws and overfitting of the training dataset. Instead, the study used an independent test set to compare the suggested architecture to state-of-the-art approaches and found that several highlighted bias and overfitting concerns are minimized.

Even though the proposed deep learning architecture provides the best performance with the best feasible setup, it highlights the difficulties in comparing and interpreting the results of various deep learning algorithms. While deep learning algorithms based on chest imaging data have shown promising results in the past, the tests imply that a more extensive, more comprehensive database, with less bias, is required for building tools that may be used in real-world clinical situations.

Using a convolutional neural network is proposed to use chest radiographs to detect COVID-19 positive individuals. Previous research has shown that COVID-19-positive patients’ lung X-rays have precise characteristics. This is a valid method for testing patients since X-ray inspection of suspect positive individuals is more accessible than PCR [97]. With a classification accuracy of 99.45% (training accuracy of 99.70%), a sensitivity of 99.30%, and a specificity of 99.40% obtained from 820 chest radiographic images (excluding data augmentation) collected from three databases, this model has proven to be a reliable COVID-19 detector.

Another machine learning algorithm is demonstrated using a SVM classifier, trained using a combination of deep convolutional and handcrafted features taken from X-ray chest scans [98]. This combination of characteristics is used to distinguish between healthy people with common pneumonia and COVID-19 patients. A regular convolutional neural network and a SVM, trained using handcrafted features, are used to compare the performance of the combined feature strategy discovered. Combining these features in the innovative framework enhances classification performance compared to applying convolutional and handcrafted features separately. The classification problem attained a 0.988 accuracy with the combined technique, compared to 0.963 and 0.983 accuracies with SVM and CNN, respectively, for handcrafted features.

DenResCov-19 is a new deep-learning network that can offer reliable classification results in multi-class lung disorders. The suggested model was tested on three distinct published datasets with four classes: COVID-19 positive, pneumonia, tuberculosis, and healthy individuals [99]. It also addressed the class imbalance issue by appropriately combining the datasets (except for DXR4, where the dataset is imbalanced in COVID-19 positive cases due to a limited number of available images).

As a result of the experimental research, the proposed model has a positive generalization and robust behavior. According to the proposed analysis, their network has better classification accuracy than state-of-the-art networks like ResNet-50, DenseNet-121, VGG-16, and Inception-V3. In addition, the proposed network would be able to provide the results of the well-balanced AUC-ROC and F1 metrics that have been confirmed. In most situations, our network’s detection points from heatmaps match the expert radiologist’s detection points. To summarize, they created a pre-screening fast-track decision network based on CXR images to detect COVID-19 and other lung diseases [99].

An approach based on pre-trained deep neural networks is shown, which has achieved state-of-the-art performance for the job at hand, i.e., 99.60% accuracy, by utilizing a cyclic generative adversarial net (CycleGAN) model for data augmentation [100]. A dataset of 3163 photos from 189 patients was also collected and labeled by clinicians to test the approach. Unlike previous datasets, standard data of persons having COVID-19 disease was collected rather than data from other disorders, and this database was made publicly available.

The suggested study includes basic measures such as grouping and clustering in the classification report [101]. The proposed methods are evaluated using J48 and Simple K Means analyses. The simulation tests the right diagnosis and confirms the accuracy figures of 99.63% for Classifying Date and 96.27% for Classifying State. Highlights are finely extracted for arrangement, and parameters are generated to create a Weka class. In structured tree decision, J48 percept is supplied ostensible information and evaluates each perceptron to send the best findings to each other.

Deep Learning has proven to be an effective way of extracting high-dimensional information from medical photos. In this paper, the state-of-the-art Convolutional Neural Network Mobile Net is used and trained from the ground up to evaluate the value of the retrieved features for the classification job [102]. Mobile Net v2, which has been shown to deliver impressive performance in related tasks, is trained using a large-scale dataset of 3905 X-ray images corresponding to six diseases.

This results both in discriminating the X-rays between the seven classes and between COVID-19 and non-COVID-19, and training the CNNs from scratch surpasses the other transfer learning strategies. Classification accuracy of 87.66% is attained between the seven classes. Furthermore, this approach has a 99.18% accuracy, 97.36% sensitivity, and 99.42% specificity in the field when COVID-19 was found to be present. The findings show that training CNNs from scratch could reveal essential biomarkers associated with, but not limited to, the COVID-19 disease. At the same time, the highest classification accuracy suggests that the X-ray imaging potential should be investigated further [102].

Using deep features, the help vector gadget distinguishes corona impacted X-ray images from others. Clinical practitioners can employ the approach to discover COVID-19-infected patients early. With COVID-19, the proposed technique of multi-level thresholding plus SVM showed good accuracy in classifying the infected lung [103]. All the images were the same size and saved in JPEG format with 512 × 512 pixels. The lung classification’s average sensitivity, specificity, and accuracy, utilizing the suggested model findings were 95.76, 99.7, and 97.48%, respectively.

Using a convolutional neural network and the pre-trained DenseNet201 model, a novel deep transfer learning model for COVID-19 disease has been developed [104]. The proposed model classifies chest CT scans as having 99.82, 96.25, and 97.4% training, testing, and validation accuracy, respectively. In comparison to specific well-known deep transfer learning models, the DenseNet201-based CNN performs much better, according to comparative evaluations.

The proposed model achieves 97% accuracy, but the accuracy of VGG-16 and Resnet152V2 is 96 and 95%, respectively. Compared to competitive models, the proposed approach demonstrated a 1% improvement. When applying the proposed method to an enormous population, however, the 1% performance advantage can save many individuals’ lives. The proposed methodology can improve the COVID-19 testing procedure because CT scans are available in most medical institutes. As a result, the proposed model can be used instead of several COVID-19 testing kits [104].

For coronavirus identification utilizing deep features and the J48 method, the chest X-ray pictures utilized for simulation purposes were acquired from GitHub and Kaggle sources. The extraction is conducted with the use of 11 pre-trained CNN models that have been separately supplied for J48 classification [105]. In addition, a statistical study is conducted to choose the optimal classification pattern. The statistical performance of the ResNet101 plus J48 classification model outperforms the other 10 competing models.

As a result, the proposed classification model’s accuracy for detecting COVID-19 disease is 98.54%. An optimized convolutional neural network model (ADECOCNN) was presented to distinguish between infected and uninfected patients. The ADECO-CNN approach is also compared to the VGG19, Google Net, and ResNet models, which are based on convolutional neural networks (CNNs). The ADECO-CNN-optimized CNN model can categorize CT images with 99.99% accuracy, 99.96% sensitivity, 99.92% precision, and 99.97% specificity, according to extensive testing [105].

Garg et al. proposed a different study type [106]. This study designed, examined, and compared, using 20 varieties of convolutional neural networks for COVID-19 detection, including EfficientNet-B5, DenseNet169, InceptionV3, ResNet50, VGG16, and others. The examined models were applied using 4173 CT images of lung tissues. As a result of this study, EfficientNetB5 was considered the best model performance with about 98% accuracy and sensitivity. This model has a higher performance and the smallest size compared to the other 21 models. Table A1 presents a summary of the existed ML and DL systems for COVID-19 detection and classification.

## 7. Conclusions

COVID-19 is a high severity disease that has spread widely over the world. Artificial intelligence systems using medical images have played a significant role in COVID-19 diagnosis. This paper discussed the most efficient and accurate AI systems for COVID-19 diagnosis using X-ray or CT scan chest images and illustrated the range of machine learning or deep learning techniques used for the detection and classification of the COVID-19 virus. The AI platforms, image augmentation, and image segmentation techniques have been briefly reviewed and discussed. Two types of images (X-ray and CT scan images) were covered in this literature to demonstrate the effectiveness of AI models for COVID-19 diagnosis.

Two main categories have been taken in order to filter the studies; the first one is using chest images, either X-ray or CT scan, whereas the other criteria were using machine learning, including deep learning algorithms. Some studies used data augmentation and segmentation which can be recognized as a valuable additive that enriches this study to be more detailed and comprehensive. Machine learning algorithms include deep learning algorithms, either the pre-defined, the improved, or the optimized ones. The Table A1 in Appendix A summarizes these studies, which are related to classification by focusing on each study’s methodology and performance.

In summary, many studies have been proposed for COVID-19 detection and classification with promising results. Screening for COVID-19 can help in early detection and aid radiologists in their diagnosis efforts. In addition, more research can be developed to predict the severity of the disease, which is very important in order to estimate the need for ICU and to make clinical decisions regarding disease treatment, therefore, reducing the load on hospitals and health care centers.

## Figures and Tables

**Table 1:** Image augmentation settings.

Method	Setting
Rotation angle	10
Width shift	0.2
Height shift	0.2
Horizontal flip	True

## Data Availability

Not applicable.

## Appendix A

**Table A1:** Summary of existing AI systems for COVID-19 detection.

Study	Year	Data Set	Methodology	Accuracy
A multi-dilation convolutional neural network for automatic COVID-19 and other pneumonia detection from chest X-ray images with transferable multi-receptive feature optimization [75]	2020	5856 X-ray images	A deep neural network architecture namely CovXNet.	97.4% COVID-19/Nomal 96.9% COVID-19/Viral pneumonia 94.7% COVID-19/Bacterial pneumonia 90.2% multiclass COVID-19/normal/ Vral/Bacterial pneumonias
COVID-19 detection in radiological text reports integrating entity recognition [76]	2021	CT Scan 295 anonymous CT scan reports	ML model NER system Five statistical parameters.	90%
Deep-chest: Multi-classification deep learning model for diagnosing COVID-19, pneumonia, and lung cancer chest diseases [77]	2021	33,676 X-ray and CT images	CNN and recurrent neural network (RNN)	The VGG19+CNN model achieved 98.05% accuracy (ACC)
COVID-19 cough classification using machine learning and global smartphone recordings [83]	2021	Data set	CNN	95.3%
Automatic detection of COVID-19 using pruned GLCM-Based texture features and LDCRF classification [78]	2021	X-ray images 2300	CAD methodology segmentation	95.88%
Diagnosis and detection of infected tissue of COVID-19 patients based on lung X-ray image using convolutional neural network approaches [79]	2020	X-ray images 682	Deep Neural Network (DNN) and Convolutional Neural Network (CNN)	CNN (93.2%) DNN 83.4%
Automatic method for classifying COVID-19 patients based on chest X-ray images, using deep features and PSO-optimized XGBoost [106]	2021	X-ray images 5586	Extreme Gradient Boosting (XGBoost) optimized by particle swarm optimization (PSO).	98.71%
COVID-19: Automatic Detection of the Novel Coronavirus Disease From CT Images Using an Optimized Convolutional Neural Network [107]	2021	CT Scan	Optimized Convolutional Neural Network	95.7%
Automatic detection of COVID-19 from chest radiographs using deep learning [108]	2020	X-ray images 1428	Deep Learning model	96%
Deep learning approaches for COVID-19 detection based on chest X-ray images [82]	2020	200 X-ray images	Deep Convolutional Neural Network	91.6%
Automatic diagnosis of COVID-19 disease using deep convolutional neural network with multi-feature channel from respiratory sound data: Cough, voice, and breath [84]	2021	4.5 k samples/web-based application. 2.5 k samples/ android based application	Multichannel Deep Convolutional Neural Network (DCNN)	80% accuracy for respiratory-based sound classification 62% for audio-based classification
EMCNet: Automated COVID-19 diagnosis from X-ray images using convolutional neural network and ensemble of machine learning classifiers [109]	2020	400 X-ray images	EMCNet	98.91%
A combined deep CNN-LSTM network for the detection of novel coronavirus (COVID-19) using X-ray images [86]	2020	613 X-ray images	Deep CNNLSTM network	99.4%
Deep Net Model for Detection of COVID-19 using Radiographs based on ROC Analysis [87]	2020	100 X-ray images	CNN	98%
The Role of Artificial Intelligence in Management of Critical COVID-19 Patients [110]	2020	CT Scan	AI	-
Detection of coronavirus Disease (COVID-19) based on Deep Features and Support Vector Machine [88]	2020	127 X-ray images	ResNet plus SVM model	98.66%.
An automatic COVID-19 CT segmentation based on U-Net with attention mechanism [89]	2020	100 CT Scan	U-Net based segmentation	-
A Critic Evaluation of Methods for COVID-19 Automatic Detection from X-ray Images [111]	2020	108,948 X-ray images	-	92%
Comparative study of deep learning methods for the automatic segmentation of lung lesion, and lesion type in CT scans of COVID-19 patients [90]	2020	1103 CT Scan	Twelve deep learning methods	-
Automatic Deep Learning System for COVID-19 Infection Quantification in chest CT [91]	2020	240,270 CT Scan	CNN	-
Improving Coronavirus (COVID-19) Diagnosis using Deep Transfer Learning [92]	2020	19,200 X-ray	CNN	98.7%
Fully automatic deep convolutional approaches for the analysis of COVID-19 using chest X-ray images [93]	2020	5856 X-ray	CNN	0.97%
Classification of COVID-19 from Chest X-ray images using Deep Convolutional Neural Networks [94]	2020	315 X-ray	Deep Convolutional Neural Networks	98%
Automatic Detection of COVID-19 Infection from Chest X-ray using Deep Learning [95]	2020	X-ray	Deep Convolutional Neural Network	93%
An Automatic Computer-Based Method for Fast and Accurate COVID-19 Diagnosis [96]	2020	195 CT Scan	CNN	92.5%
Challenges of Deep Learning Methods for COVID-19 Detection Using Public Datasets [97]	2020	CT scan	Deep Neural Network	98-99%
Automatic COVID-19 Detection from chest radiographic images using Convolutional Neural Network [98]	2020	5740 X-ray	CNN	99.45%
Classification of COVID-19 X-ray Images Using a Combination of Deep and Handcrafted Features [99]	2021	5143 X-ray/CT Scan	SVM &CNN	98.8%
DenResCov-19: A deep transfer learning network for robust automatic classification of COVID-19, pneumonia, and tuberculosis from X-rays [100]	2021	3883 X-ray/CT Scan	Deep Learning Network	86.4%
Automatic Diagnosis of COVID-19 from CT Images using CycleGAN and Transfer Learning [101]	2021	1766 CT Scan	CycleGAN	99.60%
An Automatic Classification of COVID with J48 and Simple K-Means using Weka [102]	2020	-	k-Means	99.63%
Extracting Possibly Representative COVID-19 Biomarkers from X-ray Images with Deep Learning Approach and Image Data Related to Pulmonary Diseases [103]	2020	3905 X-ray	Convolutional Neural Network	99.18%
Automatic X-ray COVID-19 Lung Image Classification System based on Multi-Level Thresholding and Support Vector Machine [104]	2020	X-ray	SVM	97.48%
Classification of the COVID-19 infected patients using DenseNet201 based deep transfer learning [105]	2020	2492 CT Scan	CNN	99.82%
ADOPT: automatic deep learning and optimization-based approach for detection of novel coronavirus COVID-19 disease using X-ray images [106]	2021	50 X-ray	11 different convolutional neural network-based (CNN) models	98.54%
COVID-19: Automatic Detection of the Novel Coronavirus Disease From CT Images Using an Optimized Convolutional Neural Network [107]	2021	CT Scan	Optimized Convolutional Neural Network	95.7%
An intelligent tool to support diagnosis of COVID-19 by texture analysis of X-ray images. Research on Biomedical Engineering [112]	2020	6309 X-ray	IKONOS	89.78%
An automatic approach based on CNN architecture to detect COVID-19 disease from chest X-ray images. [113]	2020	8830 X-ray	CNN	99.32% for binary class and 97.55% for multi-class
Automatic COVID-19 detection using exemplar hybrid deep features with X-ray images [114]	2021	11,104 X-ray	COVID-19FclNet9 (CNN optimized)	99.64%
An integrated feature frame work for automated segmentation of COVID-19 infection from lung CT images [115]	2020	80 X-ray/CT	DNN Model	-
Automatic COVID-19 CT segmentation using U-Net integrated spatial and channel attention mechanism [116]	2020	473 CT	U-Net Model	83.1%
Transfer learning-based automatic detection of coronavirus disease 2019 (COVID-19) from chest X-ray images [117]	2020	348 X-ray	Visual Geometry Group (VGG)-16, VGG-19, MobileNet, and InceptionResNetV2 (CNN optimized)	>90.0%
Deep convolutional neural networks for COVID-19 automatic diagnosis [118]	2021	1954 X-ray	CNN	99%, 99.12%, and 99.29% for ResNet18, ResNet50, and ResNet101, respectively.
Optimized genetic algorithm-extreme learning machine approach for automatic COVID-19 detection [119]	2020	188 X-ray	Optimized Genetic Algorithm-Extreme Learning Machine (OGA-ELM)	100.00%
Automatic evaluation of the lung condition of COVID-19 patients using X-ray images and convolutional neural networks [120]	2021	185 X-ray	CNN	96%
Cascaded deep learning classifiers for computer-aided diagnosis of COVID-19 and pneumonia diseases in X-ray scans [121]	2020	306 X-ray	CNN: VGG ResNe	99.9%
Evaluation of deep learning-based approaches for COVID-19 classification based on chest X-ray images [122]	2021	760 X-ray	DCNN	98.69%
Multi-task contrastive learning for automatic CT and X-ray diagnosis of COVID-19 [123]	2021	4758 CT 5821 X-ray	Contrastive Multi-Task Convolutional Neural Network (CMT-CNN)	CT (5.49–6.45%) X-ray (0.96–2.42%)
Multi-task deep learning based CT imaging analysis for COVID-19 pneumonia: Classification and segmentation [124]	2020	1369 CT	Multitask Deep Learning model	97%
Automatic detection of coronavirus disease (COVID-19) using X-ray images and deep convolutional neural networks [125]	2021	7065 X-ray	DCNN	99.7%
Deep transfer learning with apache spark to detect COVID-19 in chest X-ray images [126]	2020	320 X-ray	Deep Transfer Learning (DTL) Using (CCN)	99.01% pre-trained InceptionV3 model 98.03% ResNet50 model
Implementation of convolutional neural network approach for COVID-19 disease detection [127]	2020	4576 X-ray	DCNN	98.92%
A novel approach of CT images feature analysis and prediction to screen for corona virus disease (COVID-19) [128]	2020	51 CT	Composed Hybrid Feature Selection (CHFS) and Optimizes Genetic Algorithm (OGA)	96.07%
Automatic Classification Approach for Detecting COVID-19 using Deep Convolutional Neural Networks [129]	2020	1140 X-ray	DCNN	92.54%, precision: 93.05%, recall: 92.81%, F1-score: 92.83%, specificity: 97.47%
Artificial intelligence distinguishes COVID-19 from community acquired pneumonia on chest CT. Radiology [130]	2020	4356 CT	COVNet	95%
A machine learning-based framework for diagnosis of COVID-19 from chest X-ray images [131]	2021	500 X-ray	Logistic Regression (LR) and Convolutional Neural Networks (CNN)	95.2–97.6%
Deep learning-based meta-classifier approach for COVID-19 classification using CT scan and chest X-ray images [132]	2021	8055 CT 9544 X-ray	CNN	99.48%
Rapid identification of COVID-19 severity in CT scans through classification of deep features [133]	2020	729 CT Images	DNN (Deep Neural Network)	95.34%
Machine-learning classification of texture features of portable chest X-ray accurately classifies COVID-19 lung infection [134]	2020	250 CT Images	Deep Learning	-
Automatic classification between COVID-19 pneumonia, non-COVID-19 pneumonia, and the healthy on chest X-ray image: combination of data augmentation methods [65]	2020	1248 X-ray images	Conventional Neural Network/data augmentation	>90%
The study of automatic machine learning base on radiomics of non-focus area in the first chest CT of different clinical types of COVID-19 pneumonia [135]	2020	219 X-ray	Auto ML	95%
A novel hand-crafted with deep learning features based fusion model for COVID-19 diagnosis and classification using chest X-ray images [136]	2021	516 X-ray	FM-HCF-DLF model [Optimized CNN]	94.08%
Classification of COVID-19 patients from chest CT images using multi-objective differential evolution–based convolutional neural networks [137]	2020	CT Images	CNN +ANN+ ANFIS	92% proposed 91.7% CNN 91.4% ANFIS 89.5% ANN
Densely connected convolutional networks-based COVID-19 screening model [138]	2021	11,494 CT Images	Densely Connected convolutional networks (DCCNs) [Optimized CNN]	98.83%
End-to-end automatic differentiation of the coronavirus disease 2019 (COVID-19) from viral pneumonia based on chest CT [139]	2021	448 CT Images	Large-scale bidirectional generative adversarial network (BigBiGAN) architecture	92%
Toward real-time and efficient cardiovascular monitoring for COVID-19 patients by 5G-enabled wearable medical devices: a deep learning approach [140]	2021	-	Convolutional Neural Networks and long short-term memory networks model	99.29%
Within the lack of chest COVID-19 X-ray dataset: a novel detection model based on GAN and deep transfer learning [141]	2020	307 X-ray	GAN and Deep Transfer Learning	99.9%
Automatic detection of COVID-19 infection using chest X-ray images through transfer learning [142]	2021	194 X-ray	Different architectures of Convolutional Neural Networks (CNNs)	98.5%
Automatic COVID-19 lung infected region segmentation and measurement using CT scans images [143]	2020	275 CT	Automated tool of segmentation and measurement	98%
Automatic detection of COVID-19 from chest X-ray images with convolutional neural networks [144]	2021	165 X-ray	CNN	97.56%
Auto-diagnosis of COVID-19 using lung CT images with semi-supervised shallow learning network [145]	2021	2482 CT	CNN optimized	-----
A deep learning-based COVID-19 automatic diagnostic framework using chest X-ray images [146]	2021	6273 X-ray	Deep learning algorithm-based model	97.11%
Performance evaluation of the NASNet convolutional network in the automatic identification of COVID-19 [147]	2020	240 X-ray	Neural Architecture Search Network (NASNet)	97%
A weakly-supervised framework for COVID-19 classification and lesion localization from chest CT [148]	2020	530 CT images	DeCoVNet	97.6%
Classification of COVID-19 in chest X-ray images using DeTraC deep convolutional neural network [149]	2021	196 X-ray images	DeTraC deep CNN	93.1%
Novel artificial intelligence algorithm for automatic detection of COVID-19 abnormalities in computed tomography images [150]	2021	1581 CT images	artificial intelligence (AI) algorithm	92.0%
CCBlock: an effective use of deep learning for automatic diagnosis of COVID-19 using X-ray images [151]	2020	1828 X-ray images	enhancement of the classical visual geometry group (VGG) network	95.34%
An effective deep residual network-based class attention layer with bidirectional LSTM for diagnosis and classification of COVID-19 [152]	2020	X-ray images	work (ResNet) based Class Attention Layer with Bidirectional LSTM called RCAL-BiLSTM for COVID-19 Diagnosis	94.88%
COVID-caps: A capsule network-based framework for identification of COVID-19 cases from X-ray images [57]	2020	X-ray images	Capsule Networks	95.7%
Automated detection of COVID-19 cases using deep neural networks with X-ray images [153]	2020	127 X-ray	Deep Neural Networks	98.08%
COVID-19: automatic detection from X-ray images utilizing transfer learning with convolutional neural networks [51]	2020	1427 X-ray images	CNN	96.78%
An open-source COVID-19 CT dataset with automatic lung tissue classification for radiomics [154]	2021	62 CT images		----
Explainable artificial intelligence-based edge fuzzy images for COVID-19 detection and identification [155]	2022	5888 X-ray	Fuzzy CNNs	95%
Ensemble Deep Learning and Internet of Things-Based Automated COVID-19 Diagnosis Framework [156]	2022	12,146 CT scan	Deep learning and Internet of Things	98.98%
Explainable Machine Learning for COVID-19 Pneumonia Classification With Texture-Based Features Extraction in Chest Radiography [157]	2022	5222 X-ray	XGBoost (XGB) and Random Forest (RF).	82%
A COVID-19 CXR image recognition method based on MSA-DDCovidNet [158]	2022	5863 X-ray	multi-scale spatial attention mechanism with a convolutional neural network model (MSA-DDCovidNet)	97.962%
